# *Lactobacillus pentosus* Increases the Abundance of *Akkermansia* and Affects the Serum Metabolome to Alleviate DSS-Induced Colitis in a Murine Model

**DOI:** 10.3389/fcell.2020.591408

**Published:** 2020-10-21

**Authors:** Yong Ma, Chao Hu, Wenxin Yan, Hongmei Jiang, Gang Liu

**Affiliations:** Hunan Provincial Engineering Research Center of Applied Microbial Resources Development for Livestock and Poultry, College of Bioscience and Biotechnology, Hunan Agricultural University, Changsha, China

**Keywords:** ulcerative colitis, *Lactobacillus pentosus*, *Akkermansia*, serum metabolomics, intestinal microbes

## Abstract

*Lactobacillus pentosus* has the beneficial function of regulating the host’s immune system and plays an indispensable role in intestinal health. The purpose of this study was to investigate the specific mechanism by which *L. pentosus* relieves dextran sulfate sodium (DSS) induced ulcerative colon inflammation. We randomly divided 24 mice into three groups, which were administered either a basic diet, drinking water with 2.5% DSS (DSS), or drinking water with 2.5% DSS and intragastric administration of *L. pentosus* (DSS + *L. pentosus*). DSS was added to the drinking water on days 8 to 12, and *L. pentosus* was administered on days 12 to 19. Serum was collected for metabolomic analysis, colon length and weight were measured, and colon contents were collected to detect microbial structural composition. Compared with the DSS group, the DSS + *L. pentosus* group had significantly higher levels of indolepyruvate and pantothenic acid in the serum and significantly lower levels of 3,4-dimethyl-5-pentyl-2-furannonanoic acid and 5-oxo-6-trans-leukotriene B4. Moreover, compared with the other two groups, the DSS + *L. pentosus* group had a significantly greater abundance of *Akkermansia*. The abundance of *Akkermansia* was positively correlated with indolepyruvate and pantothenic acid levels. Therefore, *L. pentosus* can interact with *Akkermansia* to increase its abundance in the intestinal tract. This results in the production of metabolites that are beneficial for the regulation of intestinal immunity, thereby alleviating DSS-induced ulcerative colon inflammation.

## Introduction

Inflammatory bowel disease (IBD) is a chronic disease caused by the interaction of environmental, immune, genetic, and other factors (Batura and Muise, 2018). It has a significant impact on human health, and its incidence continues to increase every year (Ventham et al., 2013; Miyoshi and Chang, 2017). Ulcerative colitis (UC) is a type of IBD characterized by inflammatory disease in the colon and rectum, usually accompanied by inflammatory lesions of the colonic mucosa and submucosa (Danese and Fiocchi, 2011). In severe cases, it can cause diarrhea or even blood in the stool, and the occurrence of repeated illnesses eventually increases the risk of developing cancer (Ungaro et al., 2017). Although the specific cause of recurrent ulcerative colitis is not yet clear (Kaplan, 2015), approximately 200 genetic risk loci have been proposed for IBD. These susceptibility genes include those involved in autophagic regulation and microbial sensors that activate autophagy (Matsuoka and Kanai, 2015). Recent studies have shown that a lack of intestinal microbial protection signals promotes the expression of IBD susceptibility genes, thereby increasing inflammation (Chu et al., 2016). Therefore, we believe that remodeling the healthy gut microbial community structure will be an effective treatment strategy for colonic inflammation.

Clinical and animal model studies have shown that there are inextricable connections between the intestinal immune system and the intestinal flora (Abrams et al., 1963). For example, the transplantation of gut microbes from mice with inflammation induces inflammation in germ-free mice (Eun et al., 2014). This may occur because inflammation-related metabolites produced by specific microorganisms can activate dendritic cells and promote Th1-mediated inflammation (Devkota et al., 2012). The introduction of specific probiotics can also relieve the symptoms of inflammation and promote the development of a healthy intestinal microbial structure (Henker et al., 2008; Mardini and Grigorian, 2014). These probiotics mediate their effects by reshaping the structure of the intestinal flora, promoting the metabolic activities of beneficial flora, and improving intestinal immune activity (Marchesi et al., 2016).

An increasing number of probiotics are being used to improve human health (D’Mello et al., 2015; Park et al., 2018; Tsai et al., 2019). For example, *Clostridium butyricum CGMCC0313.1* has anti-diabetic effects, by increasing the proportion of butyric acid-producing bacteria (Jia et al., 2017). Similarly, *Bifidobacterium* strains can inhibit the accumulation of body fat, improve glucose tolerance, and ameliorate metabolic diseases (Aoki et al., 2017). However, there has been little research on the use of *Lactobacillus pentosus* to treat IBD induced by DSS. *L. pentosus* has been shown to have antifungal activity and inhibit the growth of pathogenic microorganisms (Lipińska et al., 2018). Other studies have shown that *L. pentosus* has a therapeutic effect on *Salmonella*-infected mice and it inhibits the growth of *Salmonella*, inhibits the damage caused by the invasion of intestinal epithelial cells, and improves the host’s immune function (Liu et al., 2018). Even more importantly, *L. pentosus* induces Tr1 cells to inhibit systemic inflammation (Kim et al., 2019). In intestinal epithelial cells, *L*. *pentosus* promotes the expression of TLR inhibitors, such as A20, Tollip, SIGIRR, and IRAKM, thereby inhibiting downstream MAPK and NF-κB signaling pathways, and exerting an immunoregulatory effect on intestinal epithelial cells (Kanmani and Kim, 2019). Moreover, *L. pentosus* repairs intestinal barrier damage by promoting tight junction protein expression (Zeng et al., 2020). Therefore, *L*. *pentosus* plays an indispensable role in intestinal health. In this study, we assessed the ability of *L. pentosus* to regulate intestinal microbes and investigated the specific mechanism used to alleviate DSS-induced ulcerative colonic inflammation.

## Materials and Methods

### Bacteria Preparation

The *L. pentosus* strain preserved in the Microbiology Laboratory of the College of Bioscience and Biotechnology, Hunan Agricultural University was activated using de Man, Rogosa and Sharpe medium. It was continuously cultivated for 2 days at 37°C and 120 r/min. The colonies of activated bacteria were counted using the dilution coating method to determine the concentration of the bacterial solution. The required volume of bacterial solution was centrifuged at 4,000 r/min for 10 min, the resulting supernatant was removed, and physiological saline was added to the precipitated bacteria to dilute the bacterial solution to 5 × 10^9^ cfu/mL.

### Animals and Experimental Design

**FIGURE 1:**
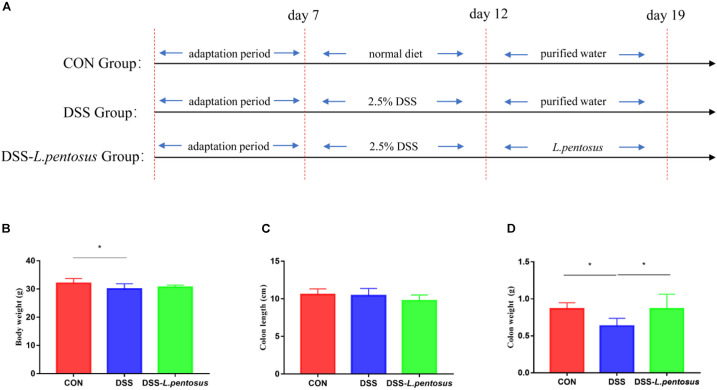
The therapeutic effects of intragastric administration of *Lactobacillus pentosus* on body weight, colon length, and colon weight. **(A)** The experimental process; **(B)** body weight; **(C)** colon length; **(D)** colon weight. Data are mean ± SD (*n* = 8) and analyzed by one-way ANOVA. *p < 0.05.

Animal experiments were performed according to the Guidelines for Care and Use of Laboratory Animals of Hunan Agricultural University. Twenty-four 8-week-old ICR mice (Hunan Silaike Jingda Co, Changsha, China), with an average weight of 18 g, were acclimated for 7 days in a sterile environment. They were then randomly allocated to three groups, with eight mice in each group. The first group (CON) was treated with a basal diet, the second group was treated with 2.5% dextran sulfate sodium (DSS) to induce ulcerative colitis, and the third group was treated with *L. pentosus* and 2.5% DSS (DSS-*L. pentosus*). During the experiment, the mice were allowed to drink and eat freely. On days 8–12 of the experiment, 2.5% DSS was added to the drinking water of the mice in the DSS and DSS-*L. pentosus* groups. *L. pentosus* was intragastrically administered to the mice in the DSS-*L. pentosus* group on days 13–19 (Figure 1). The gavage volume for each mouse was 0.1 mL. All of the mice were sampled on day 20. Serum samples were collected for serum metabolite analysis, colon samples were collected and fixed in 4% formalin, and colon contents were rapidly frozen and stored at −80°C.

### Colonic Histopathology

Colon samples fixed in 4% formaldehyde were cleared with a gradient of xylene concentrations, dehydrated with a gradient of ethanol concentrations, and finally embedded in paraffin. After sectioning, the tissue slices were placed in xylene solution I for 10 min, xylene solution II for 10 min, anhydrous ethanol solution I for 5 min, anhydrous ethanol solution II for 5 min, 95% alcohol for 5 min, 90% alcohol 5 min, 80% alcohol for 5 min, and 70% alcohol for 5 min, and then washed with distilled water. The tissue sections were then immersed in hematoxylin dye for 3–8 min; rinsed in running water; differentiated with 1% hydrochloric acid alcohol; rinsed again in running water; and finally immersed in an eosin dye solution for 1–3 min. Tissue sections were observed and photographed using a BX41 microscope (Olympus, Münster, Germany). The morphological analysis results were rated according to the scoring system presented in Table 1.

**TABLE 1:** The histologic scoring system.

Inflammation	Crypt Injury	Ulceration	Score
No significant inflammation	No injury	No ulceration	0
Neutrophilic inflammation in epithelium or lamina propria	Loss of basal one third of crypts	Two or fewer foci of ulceration	1
Inflammatory cells extending into submucosa	Loss of basal two thirds of crypts	Three or four foci of ulceration	2
Transmural inflammation	Loss of full thickness crypts	Diffuse/confluent ulceration	3

### Serum Metabolomic Analyses

Thawed serum samples were extracted with methanol, mixed with dichlorophenylalanine, and then centrifuged. The resulting supernatant was transferred to a liquid phase bottle for testing. The analyses were performed using previously described analysis platforms, chromatographic columns, and chromatographic separation conditions (Ma et al., 2019). Compound Discoverer software (Thermo Fisher Scientific, Waltham, MA, United States) was used to extract and preprocess data from the instrument, and obtain information such as the retention time, molecular weight, sample name, and peak intensity. SIMCA-P 11 was used to analyze and plot principal component analysis (PCA), Partial Least Squares Discrimination Analysis (PLS-DA), and VIP values. The retention time and molecular weight data were compared with entries in the Human Metabolome Database (HMDB) to determine the metabolite composition of the serum samples.

### 16S Ribosomal RNA Amplicon Sequencing

Microbial DNA was extracted from fecal samples using the QIAamp DNA Stool Mini Kit (Qiagen, Hilden, Germany). The V3-V4 region sequence of the extracted DNA was determined using high-throughput sequencing technology. First, using purified DNA as the template, PCR amplification was performed using the universal primers, 357F (5′-ACTCCTACGGRAGGCAGCAG-3′) and 806R (5′-GGACTACHVGGGTWTCTAAT-3′) fused with Illumina (San Diego, CA, United States) sequencing primers. The amplified products were subjected to 1.2% agarose gel electrophoresis. Successfully amplified products were run on 2% agarose gels, and the electrophoretic bands were excised to recover the DNA. The recovered PCR products were used as templates for PCR amplification (8 cycles). The adaptors, sequencing primers, and tag sequences required for sequencing on the MiSeq platform (Illumina) were added to both ends of the target fragment. An AxyPrep DNA gel recovery kit (Axygen, Alachua, FL, United States) was used to recover all PCR products, and the FTC-3000^TM^ Real-Time PCR instrument was used for fluorescence quantification. After an equimolar ratio was mixed, the library was constructed and sequenced on a MiSeq platform (PE300). Sequencing data were analyzed off-line to determine α and β diversity, using mothur (version 1.33.3). The R language was used to map species composition at different taxonomic levels and determine the differences in species abundance between groups. The abundance of the sequenced microorganisms at different taxonomic levels was uploaded to http://huttenhower.sph.harvard.edu/galaxy/ for linear discriminant analysis (LDA) and LDA Effect Size (LEfSe) analysis.

### Data Analysis

We have used SPSS 22.0 to perform bivariate Pearson correlation analysis on the data. All of the data in this study are expressed as mean ± standard deviation (SD). The data were analyzed using SPSS 22.0. The differences between the means of the experimental groups were analyzed using one-way analysis of variance and Tukey’s multiple comparison test. A *p*-value < 0.05 was regarded as a significant difference.

## Results

### *Lactobacillus pentosus* Improves Colonic Injury in Mice With DSS-Induced Colitis

After 5 days of 2.5% DSS treatment, mice were followed by 7 days of *L. pentosus* administration. The results demonstrated that the body weight of DSS group was reduced in comparison with CON group (Figure 1B, *p* < 0.05), there is no difference in colon length, but the weight of colon was decreased in DSS group in relative with CON and DSS-*L. pentosus* group (Figure 1D, *p <* 0.05).

### *Lactobacillus pentosus* Inhibits the Development of Colitis Induced by DSS

**FIGURE 2:**
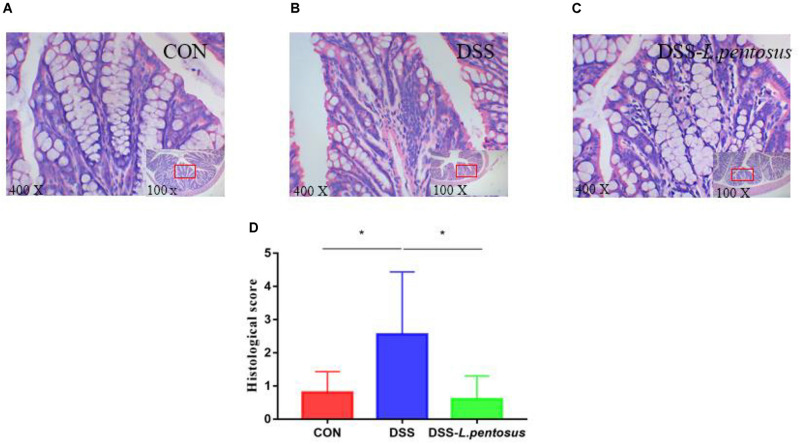
The therapeutic effect of intragastric administration of *Lactobacillus pentosus* on colon tissue damage. Images of colon morphology in the CON **(A)**, DSS **(B)**, and DSS-*L*. *pentosus* **(C)** groups under 100 × and 400 × visual fields; **(D)** Histomorphological damage scores in the three groups. Data are mean ± SD (*n* = 8) and analyzed by one-way ANOVA. *p < 0.05.

Histomorphological analysis (Figures 2A–C) showed that the colon tissue of mice in the DSS group had obvious neutrophil aggregation, goblet cell disappearance, and infiltration with other inflammatory cells. When the mice in the DSS group received *L. pentosus*, inflammatory cell infiltration was alleviated. We developed a histological scoring system (Table 1), which was used to show that histological injury was significantly greater in the DSS group than the CON and DSS-*L. pentosus* groups (Figure 2D). Thus, the intragastric administration of *L. pentosus* can relieve colonic injury induced by DSS.

### *Lactobacillus pentosus* Affects the Serum Metabolomic Profiles During Colitis

**FIGURE 3:**
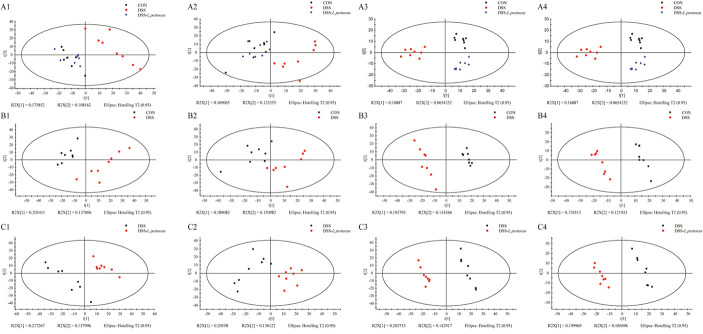
Plots of the multivariate statistical comparisons between groups. **(A1)** PCA score plot of all samples (ESI+); **(A2)** PCA score plot of all samples (ESI-); **(A3)** PLS-DA score plot of all samples (ESI+); **(A4)** PLS-DA score plot of all samples (ESI-); **(B1)** PCA score plot of CON-DSS (ESI+); **(B2)** PCA score plot of CON-DSS (ESI-); **(B3)** PLS-DA score plot of CON-DSS (ESI+); **(B4)** PLS-DA score plot of CON-DSS(ESI-); **(C1)** PCA score plot of DSS-(DSS-*L. pentosus*) (ESI+); **(C2)** PCA score plot of DSS-(DSS- *L. pentosus*) (ESI-); **(C3)** PLS-DA score plot of DSS-(DSS-*L. pentosus*) (ESI+); **(C4)** PLS-DA score plot of DSS-(DSS-*L. pentosus*) (ESI-).

**FIGURE 4:**
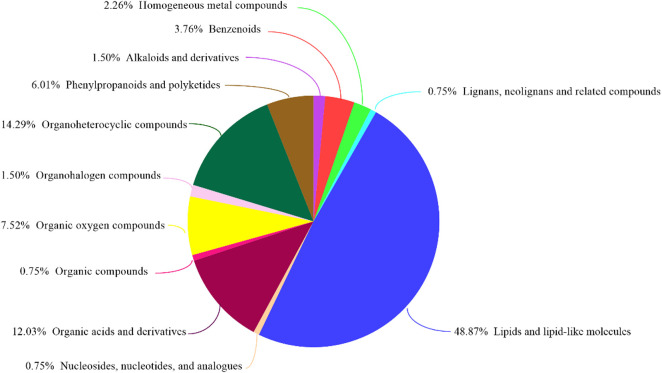
HMDB compound classification. Based on the number of metabolites, the Human Metabolome Database (HMDB) level (Superclass, Class, or Subclass) classification and metabolite percentages are displayed. The different colors in each pie chart in the figure represent different HMDB classifications, and their area represents the relative proportion of metabolites in the classification.

**FIGURE 5:**
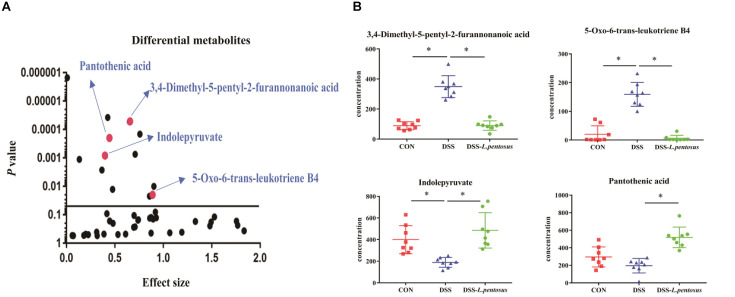
Effects of different treatments on serum metabolite levels in mice. **(A)** Volcanic map of differential metabolite levels between the DSS and DSS-*L. pentosus* groups; **(B)**Serum levels of three group metabolic products.

GC-MS was used to detect serum metabolites, and principal component analysis (PCA) to identify outliers and clusters of samples with high similarity. Partial least squares discriminant analysis (PLS-DA) was used to force the classification of each component, facilitating the identification of similarities and differences between groups. The results of PCA and PLS-DA analyses (Figure 3) showed that serum metabolite concentrations were similar within the three groups, but differed between the groups. Using PLS-DA analysis, we also identified 133 differential metabolites between the three groups, with VIP values > 1.5. These metabolites were mainly composed of lipids and lipid-like molecules (48.87%), organoheterocyclic compounds (14.29%), and organic acids and their derivatives (12.03%, Figure 4). The main metabolite differences between the three groups are shown in Table 2. Plasma levels of 3,4-dimethyl,5-pentyl,2-furannonanoic acid, 2,15-epoxy,13,14-dimethyleicosa-10,12,14-trienoic acid, alpha-dimorphecolic acid, and resolvin D5 were significantly higher in the DSS group than the CON group. However, after *L. pentosus* administration, the levels of these metabolites decreased significantly. A volcanic map illustrating the differential metabolites between the DSS and DSS-*L. pentosus* groups is shown in Figure 5A. We found that *L. pentosus* significantly reduced the production of 3,4-dimethyl-5-pentyl-2-furannonanoic acid and 5-oxo-6-trans-leukotriene B4 and other harmful substances metabolized by mice in the DSS group, and increased the serum levels of indolepyruvate and pantothenic acid (Figure 5B).

**TABLE 2:** Metabolomic changes in the plasma in the CON, DSS and DSS- *L. pentosus*.

			CON VS DSS			DSS VS DSS-*L. pentosus*		
	mz	RT (min)	VIP	*T*-TEST	Fold change	VIP	*T*-TEST	Fold change
3,4-Dimethyl-5-pentyl-2-furannonanoic acid	323.2581	7.917	2.2637	1.83195E-07	↑	2.11424	5.29157E-05	↓
12,15-Epoxy-13,14-dimethyleicosa-10,12,14-trienoic acid	313.2537	8.341	2.21032	1.18131E-06	↑	1.96774	0.010466241	↓
10,11-dihydro-20-dihydroxy-LTB4	371.2428	4.545	2.09009	2.07056E-05	↑	——	——	——
11,12-Epoxyeicosatrienoic acid	321.2424	7.41	2.08799	2.2513E-05	↑	——	——	——
Alpha-dimorphecolic acid	297.2424	7.272	1.95626	0.000177303	↑	1.79278	0.000741813	↓
Sorbose 1-phosphate	261.037	0.795	1.69084	0.002977137	↑	——	——	——
Resolvin D5	295.2268	6.881	1.63705	0.004383114	↑	1.9099	0.02057	↓
Pantothenic acid	220.1179	2.013	——	——	——	1.96618	0.000193157	↑
5-Oxo-6-trans-leukotriene B4	335.2217	6.189	——	——	——	2.15637	0.0205647	↓
11,12-Epoxyeicosatrienoic acid	321.2424	7.665	——	——	——	1.96516	3.77107E-05	↓
Indolepyruvate	204.0655	3.741	204.0655	3.741	↓	1.82665	0.000146027	↑
Phenylpyruvic acid	165.0546	3.662	——	——	——	1.61367	0.010110291	↑

### *Lactobacillus pentosus* Regulates Intestinal Microbes in Mice With Colon Inflammation Induced by DSS

**FIGURE 6:**
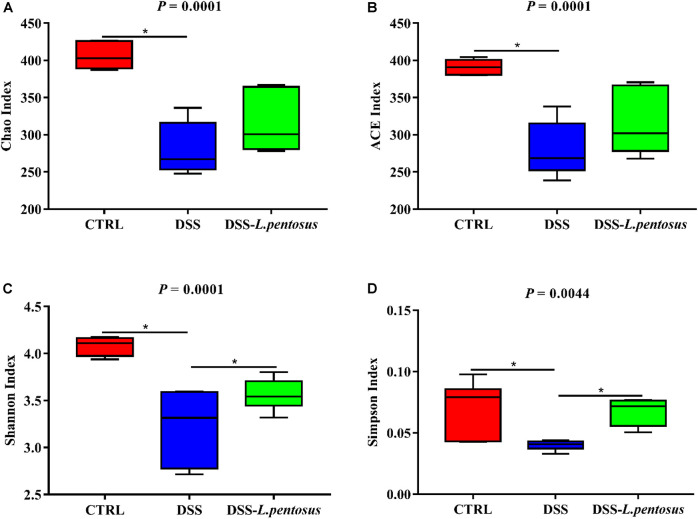
Effects of different treatments on α-diversity of mouse intestinal microbiota. **(A)** Chao index; **(B)** ACE index; **(C)** Shannon index; **(D)** Simpson index. Data are mean ± SD (*n* = 8) and analyzed by one-way ANOVA. *p < 0.05.

The 16S rRNA V3-V4 region sequences of colonic microbes were analyzed. Colonic microbe diversity was measured using the Chao index (Figure 6A), ACE index (Figure 6B), Shannon indices (Figure 6C), and Simpson index (Figure 6D). The values for all four indices were lower in the DSS group relative to the CON group (Figure 6, *p <* 0.05). Moreover, the Shannon and Simpson index values were higher in the DSS*-L. pentosus* group than the DSS group (Figures 6C,D, *p <* 0.05). Thus, *L. pentosus* administration recovered colonic microbial diversity after challenging with DSS.

**FIGURE 7:**
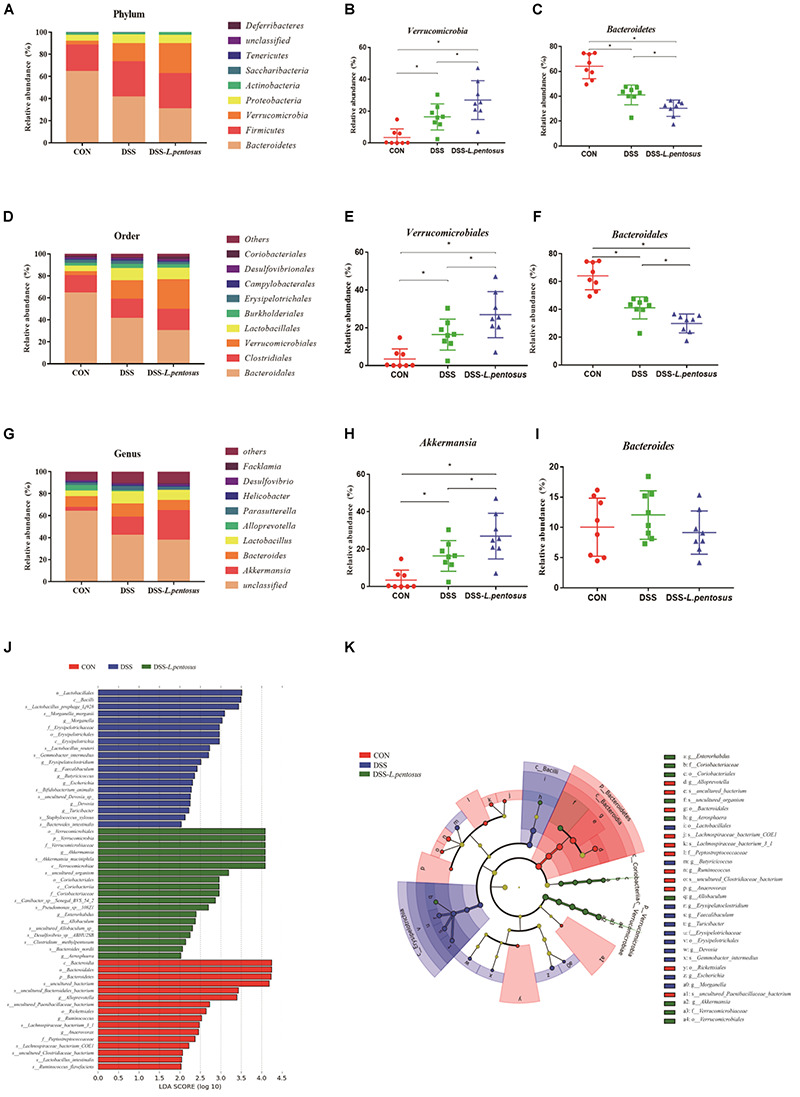
Effects of different treatments on gut microbiota in mice. **(A)** Relative abundance of gut microbiota phyla; **(B)** percentage of *Verrucomicrobia* in each sample from the three groups; **(C)** percentage of *Bacteroidetes* in each sample from the three groups; **(D)** relative abundance of gut microbiota orders; **(E)** percentage of *Verrucomicrobiales* in each sample from the three groups; **(F)** percentage of *Bacteroidales* in each sample from the three groups; **(G)** relative abundance of gut microbiota genera; **(H)** percentage of *Akkermansia* in each sample from the three groups; **(I)** percentage of *Bacteroides* in each sample from the three groups. **(J)** LDA score, an LDA score greater than 4 was considered to indicate an important contributor to the model; **(K)** LEfSe taxonomic cladogram, with different colors indicating the enrichment of certain taxa in the control (red), DSS (blue), and DSS-*L. pentosus* (green) groups. Data are mean ± SD (*n* = 8) and analyzed by one-way ANOVA. *p < 0.05.

The major bacterial phyla in the colon were Bacteroidetes, Firmicutes, Verrucomicrobia, and Proteobacteria, accounting for over 96% of colonic bacteria. In the CON, DSS, and DSS*-L. pentosus* groups, respectively, the proportions of Bacteroidetes were 64.17%, 41.09%, and 30.39%; the proportions of Firmicutes were 23.80%, 31.78%, and 31.96; the proportions of Verrucomicrobi were 3.49%, 16.45%, and 26.96%; and the proportions of Proteobacteria were 5.51%, 7.87%, and 7.56% (Figure 7A). The abundance of Bacteroidetes in the DSS-*L. pentosus* group relative to the DSS group was lower than the abundance of Bacteroidetes in the DSS group relative to the CON group (Figure 7C, *p <* 0.05). However, the abundance of Verrucomicrobia was higher in the DSS-*L. pentosus* group relative to the DSS group than in the DSS group relative to the CON group (Figure 7B, *p <* 0.05).

The ten most abundant bacterial orders are shown in Figure 7D. Bacteroidales, Verrucomicrobiales, Clostridiales, and Lactobacillales were the most abundant, accounting for more than 85% of colonic bacteria. In the CON, DSS, and DSS-*L. pentosus* groups, respectively, the proportions of Bacteroidales were 64.11%, 41.07%, and 29.85%; the proportions of Verrucomicrobiales were 3.49%, 16.45%, and 26.96%; the proportions of Clostridiales were 15.83%, 17.58%, and 19.43%; and the proportions of Lactobacillales were 5.16%, 11.16%, and 10.36%. The abundance of Bacteroidales was lower in the DSS-*L. pentosus* group relative to the DSS group than in the DSS group relative to the CON group (Figure 7F, *p <* 0.05). However, the abundance of Verrucomicrobiales was lower in the DSS-*L. pentosus* group relative to the DSS group than in the DSS group relative to the CON group (Figure 7E, *p <* 0.05).

The ten must abundant bacterial genera were selected and their percentage abundance was analyzed. The most abundant genera were *Akkermansia*, *Bacteroides*, *Lactobacillus*, and *Parasutterella*. In the CON, DSS, and DSS-*L. pentosus* groups, respectively, the proportions of *Bacteroides* were 10.04%, 12.05%, and 9.15%; the proportions of *Akkermansia* were 3.49%, 16.45%, and 26.96%; the proportions of *Lactobacillus* were 5.04%, 10.89%, and 9.27%; and the proportions of *Parasutterella* were 2.32%, 3.67%, and 2.66% (Figure 7G). The abundance of *Akkermansia* was higher in the DSS-*L. pentosus* group than in the DSS group (Figure 7H, *p* < 0.05). There was no significant difference in the abundance of *Bacteroides* among the three groups (Figure 7I, *p* < 0.05).

LEfSe analysis showed that the order *Verrucomicrobiales*, the family *Verrucomicrobiaceae*, and the genus *Akkermansia* were significantly enriched in the DSS-*L. pentosus* group, and this had a significant effect on the differences between groups (Figure 7J, *p* < 0.05). LDA was then used to estimate the magnitude of the effect of species abundance on the effect of differences between each group. Species with differential abundance between groups were identified based on LDA scores greater than 2 (Figure 7K). The abundance of species in the order *Verrucomicrobiales*, the family *Verrucomicrobiaceae*, and the genus *Akkermansia* had the most significant effects on differential bacterial abundance in the DSS-*L. pentosus* group. However, in the CON group, the abundance of species in the phylum Bacteroidetes, the class *Bacteroidia*, the order *Bacteroidales*, and the genus *Bacteroides* had the most significant effects on differential abundance, with an LDA score greater than 4.

### Correlation Between Key Microorganisms and Characteristic Differential Metabolites

**FIGURE 8:**
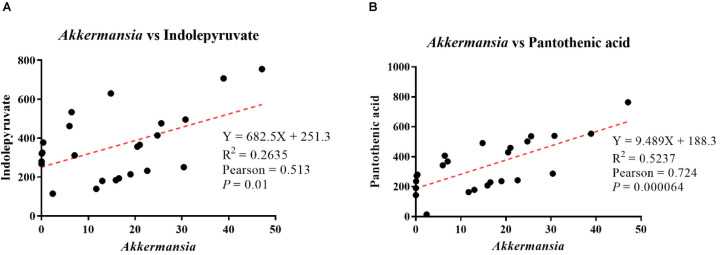
Analysis of correlations between differential metabolite levels and genus-level intestinal microbe abundance. **(A)** Correlation analysis between *Akkermansia* abundance and indolepyruvate levels. **(B)** Correlation analysis between *Akkermansia* abundance and pantothenic acid levels.

Pearson’s correlation analysis showed that *Akkermansia* abundance was positively correlated with indolepyruvate levels, with a correlation coefficient of 0.513 (*p* = 0.01, Figure 8A). Additionally, *Akkermansia* showed a positive correlation with pantothenic acid levels, with a Pearson’s correlation coefficient of 0.724 (*p* = 0.00064, Figure 8B).

## Discussion

This study explored the protective effect of *L. pentosus* against DSS-induced colon damage. We also observed that *L. pentosus* significantly regulated the microbial community structure of the mouse intestine, making *Akkermansia* the dominant flora in the colon. Through analysis of serum metabolomics, differences in indolepyruvate and pantothenic acid levels were found to be the key metabolic differences between the DSS and DSS-*L. pentosus* groups. *L. pentosus* may relieve DSS-induced ulcerative colonic inflammation by increasing the abundance of *Akkermansia* and promoting the production of indolepyruvate and pantothenic acid.

*Lactobacillus* species have been widely used as probiotics to promote intestinal health. For instance, *L. plantarum TIFN101* regulates the transcriptional pathways of tight junction and adhesion proteins, including actinin alpha-4 and metalloproteinase-2 (Mujagic et al., 2017), thereby improving intestinal barrier function. Moreover, *L. plantarum strain WCFS1* activates Toll-like receptor 2 (TLR2) signaling to promote the expression of occludin and protect the intestinal epithelial cell barrier (Karczewski et al., 2010). In our study, *L. pentosus* also protected the integrity of the intestinal structure, thus improving intestinal barrier function. Foster KR et al. showed that colonic epithelial cells interact with intestinal microorganisms to promote the stability of the intestinal microecology and thus, improve the host’s immune capacity (Foster et al., 2017). However, changes in the intestinal microbial community can disrupt the homeostasis of the colon, resulting in a loss of immune function in the intestinal epithelium (Byndloss and Baumler, 2018). These data suggest that the integrity of the intestinal structure and the stability of the internal environment of the intestinal microbial structure are key factors in maintaining immune function.

In our study, mice with DSS-induced colitis showed colon damage, and this loss of colonic barrier function caused structural changes in the gut microbiological environment. There is a dynamic balance between the host and intestinal microbes, and mechanical damage to the immune system can cause changes in the microbial community (Foster et al., 2017). Microorganisms in the colon benefit the host, because they can use complex fibers that cannot be digested by host enzymes, and fermentation can produce nutrients that the host can absorb (den Besten et al., 2013). *Bacteroides*, as the only determined sphingolipid producer among intestinal symbionts, can transport immunologically active metabolites to immune cells through outer membrane vesicles (Chu et al., 2016). Therefore, most studies have shown that IBD is associated with a significant decrease in the abundance of *Bacteroides* in the host colon (Nishino et al., 2018; Blandford et al., 2019; Brown et al., 2019). In our study, the percentage of *Bacteroides* was significantly lower in the DSS group than the CON group. However, the abundance of *Bacteroides* was significantly lower in the DSS-*L. pentosus* group than the other two groups, which may be because Verrucomicrobia became the dominant bacterial group *in vivo* after *L. pentosus* treatment, thus reducing the abundance of *Bacteroides. Akkermansia* is a symbiotic genus of the phylum Verrucomicrobia (Ansaldo et al., 2019). A large number of studies have shown that a decrease in *Akkermansia* abundance is closely related to IBD (Png et al., 2010; Rajilić-Stojanović et al., 2013). *Akkermansia* species interact with intestinal epithelial cells to promote the expression of IL-8, which regulates intestinal immunity (Drell et al., 2015). *Akkermansia* species not only induce the intestinal adaptive immune response (Ansaldo et al., 2019), but also relieve colonic mucosal damage caused by inflammation (Everard et al., 2013), by inducing the expression of tight junction proteins, including zona occludens protein-1 and occludin, and reducing the internal circulation of endotoxins (Li et al., 2016). Specifically, the anti-inflammatory properties of *Akkermansia* are shown by inhibiting the expression of pro-inflammatory factors, such as TNF-α and IFN-γ, while also stabilizing the structure of intestinal microbes (Zhai et al., 2019). Thus, treatment with *L. pentosus* increases the abundance of *Akkermansia*, promotes intestinal immunity, and preserves intestinal barrier function.

Recent studies have shown that the alteration of serum metabolite levels can be used as a potential biological indicator of gut health (Uchiyama et al., 2017). The production of certain metabolites indicates that inflammation has occurred. Most commonly, inflammation induces neutrophil aggregation and promotes the formation of neutrophil extracellular traps, and furanoid fatty acids induce NADPH oxidase and the production of reactive oxygen species in the mitochondria (Khan et al., 2018). Chemotaxis neutrophils can also secrete leukotriene B4 substances, which increase the recruitment of neutrophils (Majumdar et al., 2016). LTB4 is also packaged in multivesicular bodies and then released by neutrophils as exosomes (Majumdar et al., 2016), thereby accumulating at the site of inflammation (Németh and Mócsai, 2016). Therefore, high levels of furanoid fatty acids and LTB4 indicate the occurrence of inflammatory cell infiltration. In our study, DSS treatment significantly increased the production of 3,4-dimethyl-5-pentyl-2-furannonanoic acid and 5-oxo-6-trans-leukotriene B4, but after intragastric administration of the probiotic *L. pentosu*s, the levels of these two metabolites returned to normal. When the body is externally stimulated or when homeostasis is disrupted (such as after tissue damage or infection), tissues and immune cells produce corresponding metabolites to resolve inflammation and re-establish homeostasis (Gaber et al., 2017). When immune cells accumulate at the site of inflammation and are in a state of hypoxia, they transcribe *HIF-1α*. Indolepyruvate inhibits the HIF-1α signaling pathway induced by LPS, thereby reducing the expression levels of IL-1β (McGettrick et al., 2016). In our experiments, *L. pentosus* administration increased the production of indolepyruvate, thereby reducing the symptoms of inflammation. Ulcerative colitis is usually accompanied by the accumulation of colon stem cells and undifferentiated transit expanded cells at the base of the crypt (Papapietro et al., 2013), resulting in the inhibition of peroxisome proliferator-activated receptor (PPAR) synthesis in epithelial cells (Dubuquoy et al., 2003; Litvak et al., 2018). Pantothenic acid has been shown to continuously activate PPARs (Pourcel et al., 2020), thus reducing the risk of increased colon permeability (Ponferrada et al., 2007). However, pantothenic acid is mainly involved in metabolism in the form of coenzyme A. Coenzyme A is a ubiquitous cofactor that plays an irreplaceable role in carboxylic acid metabolism (Leonardi et al., 2005), and short-chain fatty acids have long been known to inhibit histone deacetylase and activate G-protein-coupled receptors to regulate the intestinal health (Sun et al., 2017). *L. pentosus* administration significantly increased the production of pantothenic acid, inhibited inflammation, and regulated the health of the intestine. Finally, through correlation analysis, we found that *Akkermansia* abundance was positively correlated with indolepyruvate and antothenic acid levels.

## Conclusion

Our data indicate that *L. pentosus* improves intestinal barrier function by remodeling the intestinal microbial structure and increasing the abundance of *Akkermansia*. Moreover, *L. pentosus* regulates components of serum metabolites; reduces the harmful metabolism of 3,4-dimethyl-5-pentyl-2-furannonanoic acid and 5-oxo-6-trans-leukotriene B4 caused by DSS; and promotes the metabolism of beneficial metabolites, such as indolepyruvate and pantothenic acid. Therefore, *L. pentosus* may be used as an auxiliary bacterial agent to regulate intestinal health clinically, due to its indispensable role in intestinal barrier repair.

## Data Availability Statement

The datasets presented in this study can be found in online repositories. The names of the repository/repositories and accession number(s) can be found below: https://www.ncbi.nlm.nih.gov/bioproject, PRJNA655588.

## Ethics Statement

The animal study was reviewed and approved by Animal Care and Use Committee of Hunan Agricultural University.

## Author Contributions

YM performed the study and conducted data analysis. GL designed the research. WY provided assistance for the study. HJ and CH prepared the first draft of the manuscript. All authors read and revised the manuscript.

## Conflict of Interest

The authors declare that the research was conducted in the absence of any commercial or financial relationships that could be construed as a potential conflict of interest.
